# Acute Spinal Cord Injury: A Systematic Review Investigating miRNA Families Involved

**DOI:** 10.3390/ijms20081841

**Published:** 2019-04-13

**Authors:** Enrica Pinchi, Alessandro Frati, Santina Cantatore, Stefano D’Errico, Raffaele La Russa, Aniello Maiese, Mauro Palmieri, Alessandro Pesce, Rocco Valerio Viola, Paola Frati, Vittorio Fineschi

**Affiliations:** 1 Department SAIMLAL, “Sapienza” University of Roma, 00161 Rome, Italy; enrica.pinchi@uniroma1.it (E.P.); sderrico@ospedalesantandrea.it (S.D.); raffaele.larussa@uniroma1.it (R.L.R.); aniello.maiese@uniroma1.it (A.M.); roccovalerio.viola@uniroma1.it (R.V.V.); paola.frati@uniroma1.it (P.F.); 2 IRCCS “Neuromed” – Neurosurgery Division, 86077 Pozzilli (IS), Italy; alessandro.frati@uniroma1.it (A.F.); ale_pesce83@yahoo.it (A.P.); 3 NESMOS Department – Neurosurgery Division, “Sapienza” University of Roma, 00189 Rome, Italy; mauro.palmieri10@gmail.com; 4 Forensic Pathology Institute, University of Foggia, 71122 Foggia, Italy; santina.cantatore@unifg.it; 5 Legal Medicine Division, Ospedale Sant’Andrea, 00189 Rome, Italy

**Keywords:** acute spinal cord injury, pathophysiology, clinical management, postmortem techniques, animal models, miRNAs

## Abstract

Acute traumatic spinal cord injury (SCI) involves primary and secondary injury mechanisms. The primary mechanism is related to the initial traumatic damage caused by the damaging impact and this damage is irreversible. Secondary mechanisms, which begin as early as a few minutes after the initial trauma, include processes such as spinal cord ischemia, cellular excitotoxicity, ionic dysregulation, and free radical-mediated peroxidation. SCI is featured by different forms of injury, investigating the pathology and degree of clinical diagnosis and treatment strategies, the animal models that have allowed us to better understand this entity and, finally, the role of new diagnostic and prognostic tools such as miRNA could improve our ability to manage this pathological entity. Autopsy could benefit from improvements in miRNA research: the specificity and sensitivity of miRNAs could help physicians in determining the cause of death, besides the time of death.

## 1. Introduction

Spinal cord injury (SCI) consists of a plethora of signs and symptoms resulting from a combination of different factors among which the primary impact, the subsequent cellular swelling, the continuous spinal cord compression, vascular (linked to the integrity of the arterial feeding and of the venous outflow), and intrinsic cellular mechanisms [1,2]. In recent years, there has been a progressive epidemiological increase and a trend reversal linked to a greater number of incidences in elderly people of a pathology historically linked to young age [3].

Acute traumatic SCI involves primary and secondary injury mechanisms. The primary mechanism is related to the initial traumatic damage caused by the damaging impact and this damage is irreversible. Secondary mechanisms, which begin as early as a few minutes after the initial trauma, include processes such as spinal cord ischemia, cellular excitotoxicity, ionic dysregulation, and free radical-mediated peroxidation [4,5].

Our paper is dedicated to provide a concise review in a rapidly evolving field—SCI—featured by different forms of injury, investigating the pathology and degree of clinical diagnosis and treatment strategies, the animals models that have allowed us to better understand this entity and, finally, the role of new diagnostic and prognostic tools such as miRNA and the perspectives on the future of research about SCI [6]. 

## 2. Functional and Neurological Implications of the Spinal Cord Injury

### 2.1. Traumatic SCI: Clinical Findings

**Figure 1:**
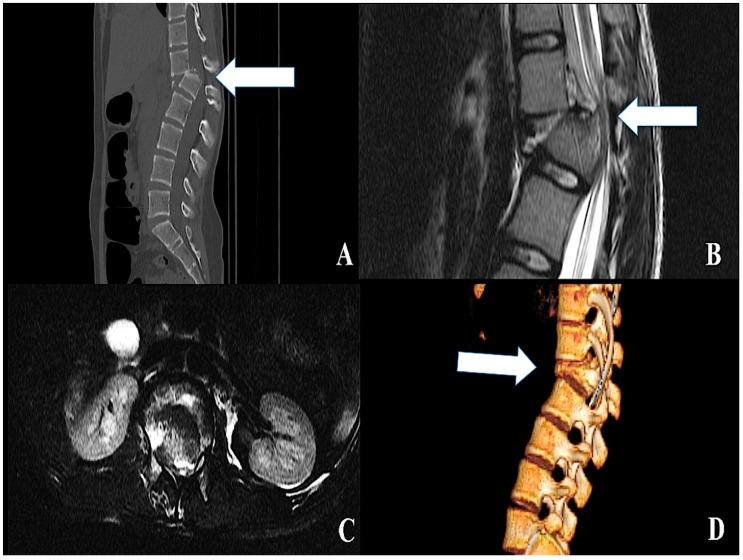
(**A**) Computed tomography (CT) scan disclosing a Thoracolumbar “C” type Spine Fracture Dislocation (arrow), with (**B**) and (**C**) complete Spinal Cord Injury (arrow). (**D**) 3D reconstruction of the CT scan disclosing the Spine Fracture Dislocation (arrow).

SCI is generally identified in clinical settings because of its neurological consequences. Each SCI level, such as Cervical (CS), Thoracic (TS), and Lumbar (LS) spine SCI, has its typical presentations, such as a lesional and sublesional syndromes and motor and sensory clinical presentations, and possible concurrent sphincter impairment (Figure 1). 

When the SCI regards the entire spinal cord, all the functions at the level and below of the lesion are lost, while if the damage is incomplete, the consequent neurological presentations depends on the horns, tracts and roots involved. In particular, in the typical lesional syndrome, damage of the posterior horns or dorsal roots could cause hyper- or hypoanalgesia, while flaccid paralysis, muscular hypotonia and hypotrophy, loss of reflexes, and fasciculations appear in the case of lesions of the anterior horns or ventral roots.

In the sublesional syndrome, the sensory clinical presentation is represented by loss of proprioception and pain, vibration, touch, and temperature sensations. The motor clinical presentation features spastic paralysis, hypertonia, and increased reflex response due to damage of the pyramidal tracts and of extrapyramidal controlling systems.

In the emergency scenario, nothing must be left to the eventuality and every patient suffering from SCI must undergo a standardized assessment, performed with dedicated evaluation tools, in order to receive an accurate prognostic information and a fine and complete neurological evaluation.

### 2.2. Evaluating Tools

Standardized tools have been created and subsequently validated in order to precisely assess and evaluate the neurological conditions of patients suffering from SCI [6]. The main advantages of such an approach include the possibility to uniform all the clinical observations in a similar fashion; to program and to obtain reliable results, both from trial concerning the clinical management; and the prognostic issues in SCI2. Each SCI patient admitted into an Emergency Department should currently undergo a standard evaluation [6,7]. Historically, the American Spinal Injury Association (ASIA) substituted, in the first half of the 1970s, the previous gold standard Frankel Evaluation Scale because of a more accurate neurological definition [8].

#### 2.2.1. Frankel Scale

The Frankel Scale synthetizes the neurological conditions of the patient affected by SCI in a purely “functional” fashion, considering the residual sensory or motor functions below the injury level; the instrument classifies five groups of patients: (A) Patients with a full motor and full sensory deficit. (B) Patients with a full motor palsy but some residual sensory function level. (C) Residual motor function but not useful for an independent life. (D) Reduced strength in concern to the motor function which is besides useful. (E) Normal neurological function. It is noteworthy that in the original paper of 1969, concerning the B and C degrees, the integrity of the sensory function was considered to be collateral, and the effective functional breakpoint is the interval between C and D scores, by which the patients are considered or not considered to be independent [8]. 

#### 2.2.2. ASIA Scale 

In respect to the Frankel Scale, the ASIA score (and subsequent revised versions) overcomes the limitations concerning the simplified “below the injury level” concept. Furthermore, the sensory function evaluation is dermatomal-based, which means it is extremely accurate for each single dermatome. The anorectal sphincter function evaluation was definitively introduced [9]. To sum up, the sensory examination evaluates 28 specific dermatomes. The examination proceeds bilaterally and includes superficial sensations (touch) and deep pain sensation (pinprick). For each dermatome, and for each side, the scores are described as follows. 0: Absent sensation. 1: Impaired or altered sensation. 2: Normal sensation [10]. The motor examination consists of bilaterally evaluating, by means of a traditional six-step (0–5) strength evaluation scale with five specific muscle groups in the upper limbs and five specific muscle groups in the lower limbs; the cervical and lumbar myotomes are responsible for the movement of the 10 most important articulations [9]. The anorectal sphincter function is directly examined in a standard fashion by means of a direct anal and bulbocavernous reflex evaluation; the possible results are presented as function (1) or absent function (0). During the spinal shock phase, in the acute traumatic SCI, ASIA score is not suitable because of the flaccid paralysis and general areflexia (including the sphincter reflexes). Although extremely accurate, the ASIA score present the major limitation of its substantial inapplicability for patients suffering from concurrent consciousness disturbances [9].

#### 2.2.3. Barthel Index

The Barthel Index [11] was introduced in 1965 with the purpose of measuring performance in activities of daily living. This tool was designed to evaluate the functional modifications due to hospitalization in rehabilitation facilities, in particular, for patients suffering from stroke. The scale has been modified in order to improve its sensibility and actually it is used for measuring the degree of independence in patients with different diseases causing impairment in daily life activities [12]. The Barthel Index is an ordinal scale composed by ten variables and three possible levels of independence for each variable. Every item is rated with a number, higher scores relate to better performances. The three possible classes for every variable are (A) the patient is completely dependent from help in the execution of that particular activity; (B) the patient can fulfill the task only with partial help from others; and (C) the patient is completely autonomous. In patients with SCI, the Barthel Index improves after systemic treatment, especially for lumbar segments [13].

#### 2.2.4. Functional Independence Measure

The Functional Independence Measure (FIM) measures the degree of disability in activities of daily living in patients hospitalized after stroke, traumatic brain injury, and SCI with a scale composed by 18 categories [14]. In particular, 13 categories analyze motor functions and five study cognitive functions [15]. Each item is evaluated with a number ranging from 1 (degree of independence in that particular daily life activity <25%) to 7 (100% of independence) and, consequentially, higher scores relate to better performances. FIM is registered at admission and at discharge, and the partial score for each time allows for the evaluation of the degree of help that the patient needs in order to accomplish basic activities. The Spinal Cord Independence Measure (SCIM) is a specific version of FIM that evaluates patients with SCI [16]. The total scores range from 0 to 100 and there are three subscales that evaluates self-care, respiratory, and sphincter management, mobility. 

## 3. Pathophysiology of the Spinal Cord Injury

### 3.1. Primary Mechanisms

**Figure 2:**
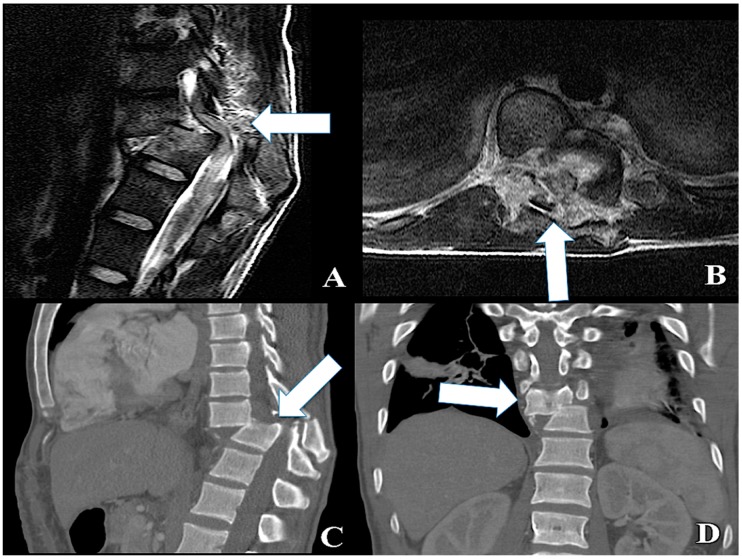
- In (**A**) and (**B**): a “C”-type Spine Fracture Dislocation with a complete Spinal Cord Injury (arrow). In (**C**) and (**D**): a CT scan of the spine disclosing a severe dislocation both on coronal and sagittal plane (arrow).

Primary mechanism consists of the direct damage on the SC tissue perpetrated by a direct trauma on the SC parenchyma. Among such possible factors are included a direct injury cause by a vertebral trauma with bony disruption and/or ligament avulsion [17], which can directly impact SC. Other direct traumatic mechanisms include a missile damage (such as in gunshot related SCI), laceration, shear damage, and distraction [1,18] (Figure 2). 

### 3.2. Secondary Mechanisms

The aforementioned list of possible mechanisms of damage causes a focal neuronal and oligodendrocytes injury resulting in a blood–spinal–barrier (BSB) interruption with increased SC parenchyma permeability and subsequent changes in the electrolytes concentration beyond the BSB, with altered sodium and calcium channel function [19,20]. Direct neurons and oligodendrocytes damage along with a damage to the microvasculature, result in a focal ischemia and a cellular swelling evolving in cellular death with release of proinflammatory factors, vasoactive peptides [21,22,23], and cytokines [1], in response to which, the resulting increased permeability includes also the inflammation cells promoting further tissue damage. Among the possible pathogenesis of such a focal ischemia, mechanical damage-related vasospasm [24], microhemorrhages [25], and focal thrombosis caused by platelet aggregation have been advocated [26,27]. A critical role has been also advocated for free radicals of oxygen [28]. Cellular excess of such species is formed, in response to focal ischemia, because of an incomplete transformation process operated by the Dismutase/Catalase enzyme system during the electron transport in the mitochondrial oxygen metabolism chain [29]. This process then result in progressive peroxidation of the cellular inner membrane causing a dysfunction of the phospholipids dependent enzymes, among which cGMP [30] and Na+/K+ ATPase, with subsequent alteration of the membrane gradients and cellular death, circularly resulting in further damage [31].

### 3.3. SCI-Related Pathways

SCI causes a deregulation of a plethora of pathways within the central nervous system [32], which will be discussed in the present section. 

The term apoptosis indicates a programmed cell death involving many cellular types, among which neurons, oligodendrocytes, and microglial cells [33,34].

It represents a major secondary pathological change of SCI. Cell death after SCI occurs after tissutal physical trauma, on one hand, and from secondary injury which enlarges the damage up to rostral and caudal zones, on the other. Moreover, inflammatory reactions greatly contribute to the development of secondary damage [35] and will be discussed later. Secondary injury following SCI plays an important role and can aggravate damage and limit the recovery and secondary mechanisms of injury, including neurogenic shock; vascular insults, such as hemorrhage and ischemia reperfusion; excitotoxicity; calcium-mediated secondary injury and fluid–electrolyte disturbances; immunologic injury; apoptosis; disturbances in mitochondrion function; and other miscellaneous processes [2]. At the RNA level, the modulation in expression of several apoptosis-related genes has been established [36]. 

The RAS genes comprises a family of membrane-bound 21-kd guanosine triphosphate (GTP)-binding proteins, which play an essential role in cell growth regulation, differentiation, and apoptosis. This effect is achieved through the interaction with MAPK (mitogen-activated protein kinase), STAT (signal transducer and activator of transcription), and PI3K (phosphoinositide 3-kinase) [37,38,39]. Usually, a deregulation in MYC (proto-oncogene, bHLH transcription factor) expression is counterbalanced by the onset of apoptotic pathways. Otherwise, cancer progression is favored [40].

SCI is known to activate an inflammatory response which starts with the alteration of the blood spinal cord barrier. Later on, peripheral immune cells infiltrate, inducing inflammatory signaling pathways [41,42]. Inflammation is responsible for secondary damage to the core and surrounding regions of the injury site [43]. Specifically, neutrophils appear at the injury site 4 to 6 h after SCI, reach a maximum at 12 to 24 h, and then disappear in 5 days [44,45]. Vascular damage in the spinal cord causes apoptosis, edema, and damage white matter [46], while endothelial cell loss and angiogenesis take place over a week post-SCI [47]. 

Astrogliosis is another typical cellular response to SCI. It implies a profound molecular and functional rearrangement of astrocytes [48] as well as an early hypertrophic neuroprotective phase, which gives way to a hyperplasic phase. Here, the formation of a glial scar hampers tissutal regeneration [49]. The first phase promotes the repair of the injured blood–brain barrier and the second one encourages gliosis process. The glial scar provides a defense and it is mainly formed by reactive astrocytes and proteoglycans [50]. The glial scar also prevents inflammatory response to exceed and limit cellular degeneration [51]. During astrogliosis, many genes are turned on in expression, such as glial fibrillary acidic protein (GFAP) and vimentin [52].

Following SCI, the injured spinal cord experiences oligodendroglia cell death and demyelination. In fact, one of the characteristics of central nervous system is its restricted capability in promoting a regenerative response. Local environmental, on one hand, and molecular features at cellular level, on the other, are responsible for this failure [53]. The possibility of a spontaneous remyelination and axonal regeneration processes is limited: a chronic and irreversible axonal damage is the final outcome [54].

The increase in reactive oxygen species (ROS) after SCI is able to recruit immune cells in the site of injury, thus enhancing secondary injury extent [55]. The endogenous antioxidant system comprises glutathione, ascorbic acid, and ROS-scavenging-related enzymes; this cellular machinery tries to fight ROS and their effects on cellular functions.

Neurogenic differentiation 6 (NeuroD6), a protein potentially involved in the development of the mammalian nervous system [56], regulates thioredoxin-like 1 and glutathione peroxidase 3. Its infusion into SCI lesions reduces the oxidization of the lesion after SCI and it is also effective in impairing apoptosis [57]. 

Neuroplasticity has been reported to occur in the adult nervous system after SCI. This process involves alterations in several molecular actors, cytokines, and growth factors, to name a few, and the sprouting of new neural connections. This ultimately leads to axon regeneration [58].

## 4. Management of the Spinal Cord Injury

### 4.1. General Management

Adequate blood perfusion and oxygen saturation and deep venous thrombosis prophylaxis are the first critical targets to achieve as per recommendation of the AANS/SCI guidelines [59,60]. Hypotension, commonly found in clinical practice following a SCI, could produce further ischemic damage. Mean arterial pressure should range between 85 and 90 mmHg for the first seven days after the SCI event. Peripheral blood saturation should be maintained at a minimum value of 90%, and low molecular weight heparin should be administered along with antithrombotic compression stockings.

### 4.2. The NASCIS Era and Its Legacy

Apart from surgery, and its role in the management of SCI, probably the most discussed treatment in the last three to four decades has been the use of Methylprednisolone sodium succinate (MP). The first observations of the early 1980s outlined a potential promising neuroprotective role of MP in SCI clinical and preclinical settings. Those observations gave rise to multiple randomized clinical trials (NASCIS I and II) that proved a statistical association with improved neurological outcomes, in a multivariate analysis in concerns to the treatment group, and the placebo group in regards to the interval between the onset of SCI and the beginning of the treatment disclosing a statistically significant better outcome when MP administration was started in less than 8 h after the trauma [61,62], with an average increase of 5 points in the ASIA score of MP group in respect to the placebo group. Such results, depicting a limited overall expected improvement, were subsequently balanced with an increased incidence of infection and wound problems along with gastrointestinal hemorrhages [63,64]; until the 2013 guidelines of AANS/CNS ruled out MP administration from the recommended measures in patients suffering from SCI [65], because of a concurrent significant evidence of potential harm [66], which nevertheless remains controversial. The current position of the AOSpine Guidelines [67] regarding the use of MP consists of administering a 24-h MP infusion to adult patients who can be treated after less than 8 h from the trauma; a 48-h MP infusion should be in any case avoided, and use of MP should be pondered in any case because of the lacking evidence of conspicuous improvements at 6- and 12-months as far as the motor scores are concerned; according to the author’s thorough literature review no statistical difference between treatment groups in the risk of complications can be unequivocally outlined. 

### 4.3. Surgical Decompression and Treatment of the Vertebral Bony Associated Lesions

A spine trauma, determining an impairment of the physiological vertebral integrity and stability by means of a ligamentous and/or bony disruption, can result in a direct trauma to the SC parenchyma related to a SCI [23]. Concerning the vertebral fractures/dislocations, only a lesser part of those conditions is associated to a neurological syndrome [67]. In such a clinical scenario, an adequate laminotomy with wide decompression of the SC, along with a restoration of the spinal stability through a spinal pedicular fixation remains, both for cervical and thoracolumbar levels, a gold standard of care [68,69,70].

In particular, the issue of the correct “timing” for the decompression procedure has been extensively debated. Currently, evidences outline that in thoracolumbar fractures, the optimal timing should not exceed 24 h to 72 h of delay in respect to the trauma [66,67,71,72]: such an early treatment is associated with a reduction of the overall complication rate, intensive care unit (ICU) stay length, infections, and the number of days of ventilator dependence [73]. Furthermore, regarding thoracolumbar fractures, an early procedure is associated with a lower incidence of bed rest-related complications, narcotic and painkiller agent use, and a faster overall recovery time [74]. 

In the clinical context of cervical spine fractures dislocation, the “timing issue” produced way more significant results: in the STASCIS prospective trial, based on clinical records of 313 SCI related to cervical spine fractures, early surgery (<24 h) was associated to a more significant probability of better neurological outcome [75]. Later evidences outline that an earlier treatment (<8 and 12 h), depending on the study, produces better functional and neurological mid- to long-term outcomes [76,77]; although such results are still controversial and deserve perhaps further unambiguous validation by evidences [78,79], which appear to intrinsically difficult to retrieve because of the vast amount of possible intervening factors, such as age, comorbidities, and absence or presence of hypotension following the trauma. 

## 5. Focused Postmortem Techniques of the Human Brain and Spinal Cord

### 5.1. Removal of the Human Brain and Spinal Cord

The brain and spinal cord may be successfully removed in several ways, depending on the pathology, especially in the presence of SCI. The goal is an intact brain ready for preliminary gross examination that has no or minimal removal artifacts.

Usually, to remove an adult brain, the head is positioned and stabilized on a block while a bitemporal incision passing near the vertex is made in the scalp down to the bone. Next, a sharp dissection peels back the skin and subcutaneous tissues posteriorly and anteriorly. The circumference of the skull is cut horizontally at the location of a crown placed on the head, with a vibrating saw or similar device. To enable realignment for postmortem reconstruction, depending on personal preference, triangular notches are cut in the skullcap; a “step cut” or an angled cut is often used. While cutting the skull cap, avoid cutting the dura to avoid penetrating the brain itself. After the cut is finished, use a hook or other wedge to separate and remove the skull cap. In adults, if the dura–brain relationship needs to be preserved, careful dissection is needed to separate the dura from the skull because it is usually tightly adhered to the inner skull. After the skull cap is removed, an incision is made in the dura from the anterior attachment. To examine the anterior skull and brain base, the brain is gently lifted and retracted posteriorly. Inspect for and record abnormalities of the anatomical structures. Dissect the olfactory bulbs from the cribriform plates, and incise the carotid arteries, third cranial nerves, and optic nerves where they exit the brain. Cut the tentorial attachment to the petrous edge and separate the posterior bony attachments of the dura and tentorium from the skull. Inspect the anterior basal posterior fossa to identify and separate the cranial nerves and vessels. Lastly, transect the medulla or upper cervical cord. To separate and remove the brain from the cranial cavity and precede to the last preliminarily steps of the examination, next, one should usually sever the vertebral arteries as low as possible. Now, the unfixed brain should be weighed (record whether this weight included the dura). Inspect the gross anatomy, and the vessels and nerves to identify and record abnormalities.

When a subarachnoid hemorrhage is present, particularly if localized, it should be dissected while fresh to avoid the consequences of fixation that usually results in a hardening of the blood mass that can make dissection very challenging. Although aneurysmal walls are usually very frail, they must be located and identified.

Once the brain is removed, the dural sinuses can be opened at the base of the skull and searched for obstructions. Indications of cerebral pressure markings can now be checked. Then, use bone forceps to remove the basal dura from the base of the skull that can then be inspected to detect fractures or other lesions. Break the dorsum sellae along its upper anterior surface using a chisel and hammer. Use scissors to cut the margins of the diaphragm while holding the diaphragm with forceps. Retract the diaphragm upward, thereby lifting the pituitary gland from its fossa; this should permit fine scissors or the point of a scalpel to reach the gland’s inferior connections, cut them to easily remove the gland.

Many different approaches and entrances to the dorsal spine are available, though these are seldom used unless there is trauma, such as from a gunshot wound or prior surgery.

In the posterior approach, the body should be placed in the prone position with blocks under the shoulders. A midline incision is made through the spinous processes, muscles are resected, and bilateral laminectomies are made using a saw. Because this method easily exposes the uppermost cervical spine and allows direct visualization of the craniocervical junction, it is recommended in cases where neck injuries are suspected or when the brain needs to be excised along with the spinal cord. Posterior dissection reveals deep contusions with blood extravasation and fractures of posterior parts of vertebral bodies also are revealed. After the spinal cord has been removed, the spinal canal can be readily examined. 

The anterior approach is the fastest and simplest way to remove the cord because the cadaver does not need to be turned over. The peripheral nerves can be followed after removal of the cord. Detailed examination of the vertebral bodies is also possible. Removal of only a part of the cord is possible, but removing the entire cord is usually preferable. 

In the anterior approach, which begins after thoracic and abdominal organs and neck evisceration, the paravertebral muscle and soft tissues are dissected laterally and, with a vibrating saw, sever the lateral vertebral processes from the high thoracic or lower cervical levels down to the lower lumbar level; avoid deep penetrations so that the spinal cord is not damaged. Incise through the high and low disks transversely to remove vertebral bodies; this will reveal the spinal cord within the dural sleeve along with the nerve roots and ganglia. Then, cut the dura vertically down to the spinal level that is visible, and incise around the dura circumferentially, while taking care not to cut the actual spinal cord. Inspect and sever the lower nerve roots, and cut across the cord itself at the caudal level. While lifting up the entire mass, cord, dura, and nerves, towards the head, sever the nerves and other connections. At this point, only the cervical nerves and the dentate ligaments connect the cervical cord to the body. Disrupt these connections by pulling on the cord so that the entire cord can be removed from below. 

**Figure 3:**
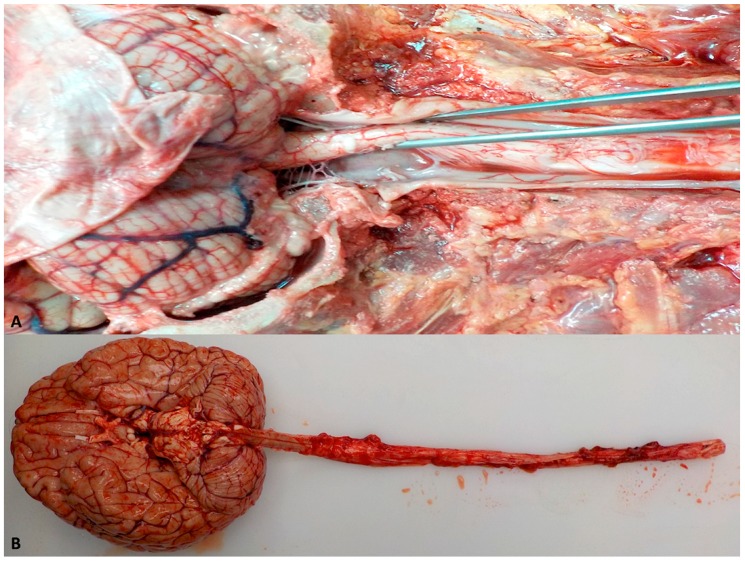
(**A**) Exposure of the cerebellum, spinal-medulla, and the spinal nerves. (**B**) Removal of the spinal cord with the brain.

Alternatively, the anterior approach also allows the complete removal of the brain with the spinal cord (Figure 3).

### 5.2. Dissection of the Human Brain and Spinal Cord

Fixation is an extremely important step in the proper examination of the brain and spinal cord as it allows for detailed anatomical studies of the nervous system. Fix the specimen, preferably with little prior handling, in a large volume of 10% formalin solution. To prevent distortion during fixation, we suspend the brain by passing a thread underneath the basilar artery in front of the pons. In the case of pontine infarcts or other lesions, a thread can be passed under the internal carotid. Alternatively, the dorsal dura can be used as an anchoring point. The specimen is not must to touch the bottom or sides of the bucket.

The options for correctly cutting a brain after fixation are many. The goal of preparing brain slices to permit abnormalities to be recognized, located anatomically, and described accurately. Consequently, description, not diagnosis, is essential. Tools reserved for brain cutting, should be stored separately from others used for general autopsy or organ dissection. The coronal brain cutting technique is probably the most frequently used and most reliable method. Fortunately, this approach is well depicted in multiple anatomic atlases; hence, anatomic references are widely available.

Before slicing, carefully examine the fixed brain externally. If the dura is present, remove it, preferably using blunt dissection; however, a scalpel can be used if needed. Then, carefully inspect the outer surfaces of the fixed brain. Next, identify each of the first nine cranial nerves bilaterally; most of the other cranial nerves, below the ninth, usually have been removed along with the brain. Now, dissect the posterior cerebral arteries at the midbrain, identify the posterior communicators, and inspect the posterior fossa arterial branches. Any lesion seen thus far should be taken. Of note, this is the optimal point to perform any special studies of cerebral cortical areas if needed because easy identification of these areas is now possible.

The cerebrum can be optimally separated from the brainstem at this time by inserting a scalpel blade lateral to one cerebral peduncle and cutting through the midbrain horizontally. Next, the midbrain section is usually cut along with the brainstem sections. By cutting serial coronal sections, the brainstem can then be examined. So that the cerebrum can be easily visualized by the pathologist, it should be oriented with the base side upwards before cutting because all the important anatomic structures are located on the base. Then, serial coronal sections are cut; each cut should be made smoothly, not sawed; start cutting with the base of the knife and end at the tip. Because sawing can crush tissue, it should be avoided. Begin the first sectioning at the temporal tips, so hydrocephalus, if present, can be seen on this section. Make sections 0.5-cm thick; thicker sections are needed in the case of massive hemorrhage and if the brain is softened.

Occasionally, for special purposes, alternative approaches to preparing sections may be required. Examples for visualizing midline abnormalities that are often better demonstrated using a midsagittal cut of the whole brain, or in order to make parasagittal or steep angular cuts to identify anatomic fiber tracts. Alternatively, the cerebellum can be cut along the horizontal plane or in along planes perpendicular to the folial orientation. A combination of both methods also can be used. Preparing sections of the brain stem and cerebellum should be consistent with the principles used for the cerebral hemispheres.

For examination of the spinal cord, after the dura has been opened along the anterior midline and the cord surface has been examined, a series of cross-sections are prepared. The dura should be left attached to the cord to keep the sectioned spinal cord and roots together. When specific radicular-level involvement has been reported premortem, the involved roots should be identified and processed separately. Use a blade to section the spinal cord at about at 1-cm intervals. Occasionally, longitudinal sections can be cut to visualize the rostral–caudal extent of a lesion, such as in traumatic contusion. However, achieving a straight plane in a longitudinal section is often difficult.

### 5.3. Selection of Tissue Blocks for Histologic Examination

**Figure 4:**
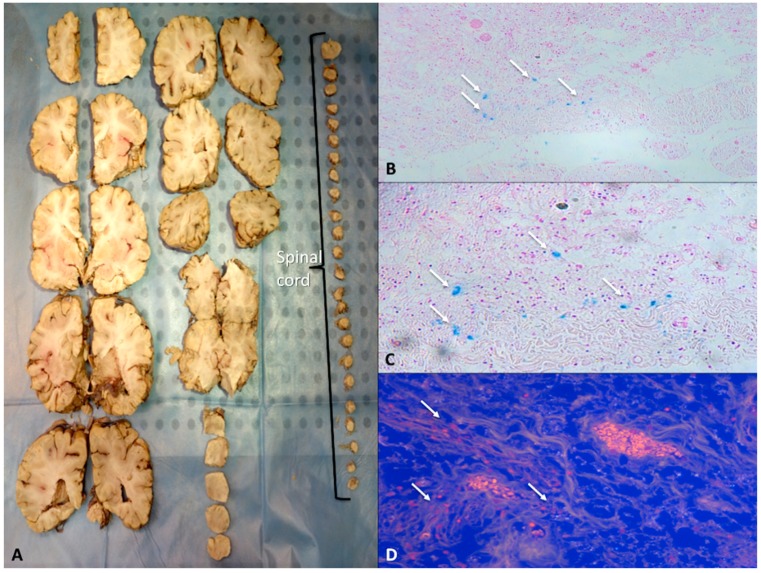
(**A**) After accurate sections, spinal cord roots and vertebral levels can be counted. (**B**) Subacute traumatic injury of the spinal cord: clearly visible, in **B** and **C**, ferrous particles deriving from hemoglobin colored by the method of Perls (arrows) (**B** ×60; **C** ×100). (**D**) In fluorescence, small hemorrhagic collections in subjects with acute cervical medullary injury (arrows) (×200).

When the lesions in the brain are obvious, selection of the appropriate blocks is simple. No universally accepted standards exist, but whatever choices are made, selection of blocks should be topographically consistent (Figure 4).

In presence of spinal cord lesions, pathologists should attempt to locate the radicular-segmental or vertebral body level of the lesion. To correctly locate the spinal levels, the dural sac and nerve root exits must be intact.

## 6. Animal Models for the Understanding of SCI

### Protocol of Study

Worldwide, animal models represent a developing research area, since they hold promise in improving human therapeutic iter. For SCI research, the need for a reliable animal model is peremptory, being possible to cause a trauma and monitor carefully all variables involved, but this does not mean that satisfactory results can be easily obtained. Ideal models should reach high levels of reproducibility, stability, and feasibility, as well as similarity to clinical SCI. Methods for reproducible and controlled SCI models have been well described [80,81], together with many behavioral outcome measures [82,83,84]. All these studies have clarified the pathophysiology of SCI, ameliorating the current perspective of functional recovery. So far, many animal models have been developed, of which murine models are undoubtedly the most rapid and least expensive ones. 

Of these, rats are most frequently used for their low cost, availability, and similarity to human SCI functional outcomes [85]. Obviously, various animal models own advantages and disadvantages, but no one can completely satisfy both research and clinical needs, although many progresses have been made in the last two decades. 

Furthermore, clinical trials have revealed the significant dissimilarity between animal and human SCI. Although quite similar in theory, the pathology of human SCI presents distinctive elements. In other words, as exciting as the findings from animal studies are, they are multifaceted and often contrasting those from clinical studies, lowering model translational impact on human outcomes [86,87]. This is probably because human SCI is heterogeneous in nature [88], besides anatomic differences between the species.

The spinal region most frequently studied through animal models is the thoracic one, whereas cervical zone is analyzed to a lesser extent. This is quite surprising, since human SCIs commonly occur at the cervical level, mainly due to motor vehicle accident and sport injury, and thus should deserve particular attention [89]. 

This is mainly due to the fact that cervical SCI can be lethal for the animal, causing impairment of respiratory functions [90,91].

SCI models can be classified on the basis of the mechanism of injury as contusion, compression, distraction, dislocation, transection, or chemical [92].

In contusion models, a transient force applied through weight-drop, electromagnetic, or compressed air instruments causes spinal cord injury [93,94], while in compression models, the spinal cord injury is achieved through prolonged compression [95]. In distraction models, traction forces extend the spinal cord with opposite trends [96]; lateral displacement of vertebra is obtained in dislocation models [97]; transection implies spinal cord severing [98], and finally, chemicals are used to dissect secondary signaling cascades caused by SCI [99,100,101].

## 7. The Role of miRNA after SCI

### 7.1. miRNAS: Stucture and Function

MicroRNAs (miRNAs) consist of endogenous, noncoding, single-stranded, 22-nucleotide-long RNAs, which are responsible for the post-transcriptional genic regulation [102]. MiRNAs are estimated to regulate more than half of all genes in the human genome [103], and increasing evidence demonstrates that a large number of miRNAs are expressed in the central nervous system [104]. Some miRNAs are involved in several neurological disorders, including traumatic CNS injuries and neurodegenerative diseases [105,106,107], thus, their role in regulating SCI-related signaling networks is supposed to be crucial. Many authors have claimed that miRNAs are potential new targets for the treatment of SCI. Since miRNAs can regulate many genes at the post transcriptional level, they are attractive candidates as upstream regulators of the secondary SCI progression [108].

### 7.2. miR-20a and miR-29b

Since miRNAs regulate a plethora of genes at the post-transcriptional level, their involvement in apoptosis regulation after SCI is not surprising. Among all, miR-20a and miR-29b have been proved to target two important mediators of neuronal apoptosis, BH3-only family genes, and myeloid cell leukemia sequence-1 (Mcl-1), belonging to Bcl-2 family [109,110,111]. An animal model of neuro-2A neuroblastoma cells has been employed to assess the involvement of miR-20a and 29b in neuronal apoptosis of SCI. The authors demonstrated that miR-20a and miR-29b mimics were able to decreased Mcl-1 expression and Bad/Bim/Noxa and Puma, respectively. The authors injected miR-20a and miT-29b inhibitors in two distinct animal groups (C57BL/6 mice) during laminectomy at the 10th thoracic spinal vertebrae (T10) [109]. As explained in the previous section (animal models), the vast majority of in vivo studies about miRNA involved in SCI opt for thoracic injuries (in particular, laminectomy at the 10th thoracic spinal vertebrae), since it is possible in this way to avoid fatal cases, on the one hand, and analyse the effects of rehabilitation exercise on gene expression, on the other. Another study showed that abnormal expression of miR20a is able to induce secondary injury in adult female mice subjected to T9-T10 laminectomy. In this study, authors injected miR20a in surgically exposed spinal cord and demonstrated miR20a to induce apoptotic neural cell death after two days of infusion. Interestingly, miR20a was able to induce a type of SCI very similar to the traumatic one. Concomitantly, miR-20a target genes, such as *Ngn1*, *Ngn2*, *DCX*, *SMAD1*, *E2F1*, *PBXO6*, *CDK2*, and *CDK4*, were reported to decrease in expression [112]. All these findings suggest that miR20a could be a potential target for therapeutic intervention after SCI. 

### 7.3. miR-223

MiR-223 is a highly conserved miRNA, which was first characterized as a myeloid-specific miRNA among all hematopoietic cells [113]. Although its function has been only partially described, miR-223 was proved to regulate granulocyte differentiation and activation [114]. The involvement of miR-223 in boosting inflammatory response through modulation of NLRP3 inflammasome activity was also demonstrated (NLRP3 inflammasome activity is negatively controlled by miR-223). MiR-223 is involved in post-SCI apoptotic pathway. Again, an in vivo model of thoracic SCI was built (in this case trauma was inflicted at thoracic vertebra 8); the injection of antagomir-223 reduced Bax and caspase-3 expression levels, ultimately reducing cell apoptosis [106]. Furthermore, the silencing of miR-223 also improved recovery after trauma, making miR-223 a candidate for the development of new therapeutic strategies for SCI. Another study involving microarrays for the detection of miRNA alteration after SCI demonstrated the increase in miR-223 levels 14 days after SCI [107]. Other reports confirmed the increase in expression of miR-223 post-SCI, although shortly after trauma (6 to 12 h after SCI) [108]. Overall, accumulating evidence indicates miR-223 as a potential therapeutic target to achieve functional recovery, angiogenesis and anti-apoptosis after SCI [115]. Besides being involved in the apoptotic pathway, miR-223 has been also linked to inflammation-related pathways after SCI. In particular, miR-223 was demonstrated to increase in response to neutrophil recruitment [116], which occurs soon after SCI [54], and its association with myeloid cell differentiation was also assessed [114]. Similarly, other authors confirmed the increase in miR-223 RNA levels 6 h, 12 h, and 3 days after SCI. They hypothesize this upregulation to be related to inflammatory reactions after SCI [117]. Furthermore, miR-223 was also proved to be involved in neurotoxicity, since its upregulation is able to silence the expression of NMDA and AMPA receptor subunits [118].

### 7.4. mir-21

The sequence of miR-21 is well conserved across species. So far, this miRNA has been mainly characterized for its implication in carcinogenesis [119]. MiR-21 has been shown to exert an antiapoptotic function: it is upregulated in the vast majority of solid cancers. In fact, its most important direct targets are apoptotic-related genes, such as PDCD4 (programmed cell death protein 4) and RECK (Reversion-inducing-cysteine-rich protein with kazal motifs). Tumor suppressor tropomyosin 1 (TPM1) has been confirmed as a target of miR-21 [120].

TPM1 and TPM2 isoforms are suppressed in malignant cells, thus being implicated in neoplastic transformation [121]. PTEN (phosphatase and tensin homologue) is probably the most well-studied tumor suppressor gene, which is inactivated by increased levels of miR-21 [122]. Finally, APAF1 (apoptotic protease activating factor-1), which is the molecular core of the apoptosome, was reported to contain a strong miR-21 binding site, prompting the authors to propose it as a direct target of miR-21 [119]. All these findings suggest miR-21 to act as an antiapoptotic factor, which would protect neural cells from death through the silencing of proapoptotic molecules [123].

The abnormal expression of miR-21 has been demonstrated in many injury models of SCI. For example, in a rat model of T10 contusive SCI, the upregulation of miR-21 has been established by microarray analysis [105]. The same results were reported in another microarray analysis involving rat thoracic models of SCI (contused at T8). This increase in expression was also observed seven days postoperation [106]. Another study implying rat models subjected to laminectomy at T12-T13 level reported an initial increase in expression of miR-21, followed by a deep switching off. The increase in expression of miR-21 was hypothesized to exert a protective effect for neural cells [124], since its silencing was reported to induce apoptosis [119,125,126]. In accordance with this study, Bhalala and colleagues reported an upregulation of miR-21 in astrocytes in the site of trauma, which was responsible for the suppression of the hypertrophic response to SCI [127]. The overexpression of miR-21 was demonstrated to impair ischemic neuronal death, prompting researchers to suggest it as a candidate for the development of new therapeutic strategies for stroke. This mechanism was demonstrated to involve Fas ligand gene (FASLG) silencing [123]. In accordance with these findings, the use of antagomiR-21 was also demonstrated to increase Fas ligand (TNF-a family) expression at day 3 after SCI [128]. A recent transcriptomic study made use of a microarray screen to identify miRNAs altered in expression after sciatic nerve injury. Among others, miR-21 showed an upregulation; furthermore, miR-21 promoted neurite outgrowth in rat dorsal root ganglion neurons, demonstrating its involvement in regenerative processes [129].

### 7.5. mir-15 and mir-16

miR-15 and miR-16 are clustered at the 13q14.3 genomic region, which is frequently deleted in high-stage tumors. Besides deletions and translocation events involving these miRNAs, their downregulation was reported in B cell of patients suffering chronic lymphocytic leukemia [130].

Members of this miR family are involved in crucial cellular pathways, such as cell division, metabolism, stress response, and angiogenesis. At the pathological level, they were demonstrated to play crucial roles in carcinogenesis, cardiovascular diseases, and neurodegenerative diseases [131]. They also act as tumor suppressor genes inhibiting BCL2 expression, and thus favoring apoptosis [132]: their increase in expression is concomitant to the decrease in expression of Bcl-2, an antiapoptotic factor [133], and the increase in expression of caspases 3, 8, and 9, the final effectors in the apoptotic pathway [134]. The crucial role of miR-16 and miR-15b on apoptosis has been investigated through multiple approaches, such as miRNA profiling assays, TUNEL staining, and annexin-V/PI labeling flow cytometry [132]. In rat models of SCI, the upregulation of miR-16 and miR-15b after injury was demonstrated to be linked to Bcl-2 silencing [135]. Furthermore, exercise after SCI increased the expression of miR-16, while miR-15b levels decreased, with concomitant change in expression of their target genes [136]. 

### 7.6. miR-124

MiR-124 is an highly conserved miRNA. Its expression is restricted to the muscle and the central nervous system. MiR-124 is almost absent in neural progenitors, while it is highly expressed in neuronal cells after differentiation [137] until becoming the most abundant miRNA in the adult brain [138]. MiR-124 is hypothesized to exert a crucial function in the differentiation of progenitor cells to mature neurons [139]. Nonetheless, miR-124 was demonstrated to promote ectoderm differentiation towards a neuronal lineage. This effect is achieved through a negative feedback loop inhibiting REST-mediated repression of neuronal genes, with concomitant shutdown in expression of non-neuronal genes [140]. In a rat model of SCI, in situ hybridization localized miR-124a expression in the gray matter. Expression assays showed a decrease in miR-124a levels 12 h after trauma lasting for a week [44]. The authors concluded that the trend in expression of miR-124a expression may reflect cell death after SCI. The expression of miR-124a was investigated in a mouse model of SCI and monitored for seven days after trauma. Expression levels of target miRNA were confirmed by in situ hybridization [117]. Results showed a decrease in expression of miR-124a after SCI. In a rat model of cervical SCI and in cell cultures, miR-124 delivery was demonstrated to reduce the activation of microglial cells, reducing MHC-II, TNF-α, and ROS production in bone marrow derived macrophages [141]. The decrease in miR-124 expression in contused animals could be attributed to the high degree of neuronal death resulting from the primary injury [117]. However, Sox2 and REST genes show upregulation in large motor neurons in which miR-124 expression has been silenced, letting researchers to hypothesize cellular dedifferentiation to be responsible for the decrease in miR-124 expression [124].

### 7.7. miR-200c

MiR-200c belongs to the miR-200 family, which regulates the epithelial-to-mesenchymal transition in various cancer types. MiR-200 levels were reported to decrease in various tumors and this event increase cancer cell aggressiveness. On the contrary, the introduction of miR-200 is detrimental for cancer cell growth. [142,143,144]. The mechanism by which miR-200c is able to induce apoptosis involves the regulation of FAP-1 [145]. In accordance with these findings, a significant increase in expression of miR-200c due to post-SCI apoptosis has been recently assessed by Yu and coworkers, concomitantly to the downregulation of Fas-associated phosphatase-1 (FAP1). Another study dealing with murine microglial cell lines reported that the upregulation of miR-200c promoted apoptosis and impaired functional recovery [145]. These findings may suggest new therapeutic strategies for recovery after SCI.

### 7.8. miR-486

MiR-486 has been recently demonstrated to silence the expression of neurogenic differentiation 6 (NeuroD6), a neuroprotective protein that triggers the expression of ROS scavenger proteins (GPX3, thioredoxin). NeuroD6 is a target of miR-486 in the motor neuron; this protein is responsible for neuronal differentiation and the oxidative stress response [146]. It induces the expression of GPx3 (glutathione peroxidase 3) and TXNL1 (thioredoxin-like 1) in SCI, which contrast ROS (reactive oxygen species) dangerous effects and inflammatory reactions [147]. In murine models of SCI, the increase in expression of miR-486 was noticed in motor neurons seven days after injury. This event was responsible for the repression of NeuroD6. The ultimate effect was the decrease in ROS scavenger proteins and increased neurodegeneration mediated by oxidative stress. This mechanism was confirmed by administration of miR-486 and silencing assays in the same animal model [147]. In fact, the infusion of miR-486 into the spinal cord of healthy animals was able to decrease the motor function and to promote neuronal death. Silencing assays were performed knocking down miR-486 in a mouse SCI model; here, functional recovery was promoted, suggesting new therapeutic avenues in clinical treatment of SCI.

### 7.9. Let-7

The Let-7 family is highly conserved and is composed by 12 members, one of which is let-7a. Various human let-7 genes map to regions which are subjected to deletion in human cancers [148]. These findings, together with others reporting let-7 downregulation in tumors [149], suggest that this family could act as tumor suppressors. In accordance with these findings, the overexpression of let-7 was able to stop cell growth in a cancer cell line [149]. Despite these evidences, the exact mechanism by which let-7 regulates cell cycle is only partially known. The antiapoptotic genes RAS [150,151], and MYC are both targeted by let-7a, which ensures their silencing in physiological conditions. For these reasons, let-7a could cooperate with miR-21 in their regulation [152]. Several members of this family, specifically let-7a, let-7d, and let-7g, were found to be downregulated after SCI [106]. The increased levels of cytokine IL-6 during the first days after SCI correlates with the decrease of its regulator, let-7a [153].

### 7.10. miR-96 and miR146a 

Mir-96 and miR-146a belong to the let-7/miR-98 family, a highly conserved family that consists of 12 members [154], and plays a key role in cell proliferation, differentiation, and oncogenesis [155]. MiR-96 and miR-146s are known to decrease after SCI [99,100]: this trend favors apoptosis through the concomitant increase in expression of their targets, proapoptotic proteins caspase3 [156], and Fas/CD95 [157]. Interestingly, an increased expression of miR-146a is observed seven days after SCI [106,146], and this event exerts its inhibitory effect on NF-κB expression [158] through a negative feedback that ultimately cause the inactivation of NF-κB pathway. 

### 7.11. miR-107

Bcl-2 is a target for many miRNAs, one of which is miR-107. A recent work made by Liu et al. demonstrated the upregulation of this miR 4 h after SCI. The authors suggested this event to cause the increase in Bcl-2 levels thus promoting apoptotic pathway. After seven days, miR-107 levels decreased in expression, inverting this trend [105].

### 7.12. miR-1

MiR-1 exerts different roles in cell proliferation and differentiation [159]. Furthermore, miR-1 is able to arrest the already initiated differentiation process of neuronal cells [160]. These characteristics render miR-1 an interesting candidate for the study of neural responses after SCI.

A significant decrease in miR1 expression after spinal cord contusion has been reported [124], although these alterations were not confirmed after SCI [161]. 

Experimental findings on SCI regarding miR-1 are controversial. In fact, upregulation after SCI was reported by Liu et al. [105], while downregulation was noticed by other authors in similar experimental conditions [106,124]. Since miR-1 plays crucial roles in sensory reactivity and inhibition of angiogenesis [162] its change in expression reported in SCI could be attributed to a vascular response to trauma [161]. Despite the fact that this hypothesis was reliable, RT-PCR assays in heart and carotid arterial tissue did not confirm an alteration in expression of miR-1, suggesting that changes in miR-1 expression after spinal cord contusion [124] were confined to the injury site rather than reflecting a systemic adaptation.

### 7.13. miR-129

Human miR-129-1 is located in the genomic regions near FRA7H: this is one of the 7q32 chromosome fragile sites that is subjected to deletion in several solid tumors. miR-129 is hypothesized to exert a tumor suppressor activity. Its downregulation was evaluated in many tumor cell lines (with respect to normal counterparts) [163,164,165,166]. Moreover, miR-129 expression appears to correlate with tumor differentiation status [167,168,169]. Together with miR-1, miR-129 presides over transcription, differentiation, and cell cycle-related processes, and may promote an aberrant mitotic phenotype at the injury site after SCI [170]. In fact, miR129 family suppresses the expression of CDK6, a G1/S phase-specific regulator, thus miR129 silencing is a permissive situation for cellular proliferation of cells arrested at the G1 phase [171]. In the study conducted by Wu in 2010, miR129-2 and miR129-1 were downregulated four days after SCI [171]. Similarly to these findings, miR-129 showed a downregulation in the injury sites of contused rat spinal cords [124].

All the discussed data about the role of miRNAs after SCI are summarized in Table 1.

## 8. The Future of SCI-Related miRNAs

In the last few years, miRNAs have attracted scientific interest in the field of forensic pathology. This is certainly due to their intriguing characteristics, such as their high resistance to external factors, their tissue/fluid-specificity, or disease-specificity. Last but not least, miRNA research is not quite expensive [172]; all the above mentioned features make miRNAs the ideal candidates for daily practice of the forensic laboratory. Here, it is the norm to work with degraded samples [173,174]. So far, forensic studies investigating miRNA expression have tested their specificity to serve as an alternative tool for body fluid identification [173,174,175,176,177,178]. However, results are often controversial. MiRNA dysregulation has been reported in almost all physio and pathological conditions, including traumatisms. Traumatic brain and spinal cord injuries (SCI) are the most common causes of disability in young adults. Several studies regarding miRNA expression in these multiple traumas have been conducted [105,179,180,181,182,183]. Therefore, the study of miRNAs may provide new insights into the molecular mechanisms of SCI. Almost all these studies deal with animal models, whereas clinical studies are rare. One exception is a study that analyzes miR-185 expression and its target—TGF-β1—in bone tissue, blood, and cerebrospinal fluid [184]. 

## 9. Conclusions

SCI is featured by different forms of injury, and after investigating the pathology and degree of clinical diagnosis and treatment strategies, animal models have allowed us to better understand this entity and, finally, the role of new diagnostic and prognostic tools such as miRNA could improve our ability to manage this pathological entity. Autopsy could benefit from improvements in miRNA research: the specificity and sensitivity of miRNAs could help physicians in determining the cause of death, besides the time of death [185]. The literature describes specific microRNAs that may provide indications for understanding some crime scene investigations and pathological processes in the cadavers. Forensic research using microRNA has been primarily used for the identification of body fluids, but its use in understanding pathological processes in postmortem pathology has not been exhaustively studied [186]. Again, it has been hypothesized that miRNAs present in vitreous humor could be a sort of “biological black box”, storing information about physiological and environmental circumstances at death. Authors support the potential forensic utility of the analysis of miRNAs in the vitreous humor in applications such as determining the time of death [187].

For all the above-mentioned reasons, miRNAs could become in the next future reliable forensic biomarkers for the diagnosis and prognosis of SCI. Specifically, those miRNAs involved in the SCI-related pathways, which were discussed earlier in the present review, could become part of the routinary clinical practice integrating current histological and immunohistochemical investigations.

## Figures and Tables

**Table 1:** Roles of different miRNA in SCI.

ID	Model	Expression	Experimental Setup	Method	Site of Injury	Targets	References
mir20a mir29b	Adult female C57BL/6 mice	mir20 upregulated mir-29b downregulated	Injection of mir20a and mir-29b in two animal groups of during SCI	Contusive	T10	downregulation of antiapoptotic myeloid cell leukemia sequence-1 (Mcl-1) and up-regulating proapoptotic BH3-only proteins.	[109]
mir-223	Adult male Sprague Dawley rats	upregulated	1, 3, 7, and 14 days after SCI	Contusive	T8	the injection of antagomir-223 reduced Bax and caspase-3 expression levels, ultimately reducing cell apoptosis	[115]
	Male C57BL/6 mice	upregulated	12 h after SCI	Compressing the cord laterally from both sides for 10 s with a number 5 forceps	T11–12	miRNA-223 may reflect inflammatory responses	[117]
	Adult male C57BL/6 mice	upregulated	from 6 to 12 h after SCI	Compressing the cord laterally from both sides for 10 s with a number 5 forceps	T11	miR-223 is expressed in neutrophils that relate to the inflammation in the epicenter after SCI, and inflammatory cytokines were also highly expressed within the same range.	[116]
mir-21	Adult female SD rats	upregulated	4 h, 1 day, and 7 days after SCI	Contusive	T10	Inflammation, oxidation and apoptosis	[105]
	Adult female Wistar rats	upregulated	1, 3, and 7 days after SCI	Contusive	T8	TPM1, PTEN [148], PDCD4 [98], proapoptotic [149]	[106]
	Male Sprague Dawley rats	upregulated	4 and 14 days after SCI	Contusive	T12–T13	Suppression of miR21 has been shown to cause apoptosis in both cortical progenitor cells and gliomas	[124]
mir-15 mir-16	Adult female Sprague Dawley rats	downregulated	12 h after SCI	Compressing the cord laterally from both sides for 10 s with a number 5 forceps	T9–T10	Target genes: proapoptotic (decreased PTEN, PDCD4 and RAS mRNA) and antiapoptotic (increased Bcl-2 mRNA). Down regulation of mRNA for caspase-7 and caspase-9 and reduced levels of caspase-7 protein.	[136]
mir-124	Male C57BL/6 mice	downregulated	12 h after SCI	Compressing the cord laterally from both sides for 10 s with a number 5 forceps	T11–12	reduce the activation of microglial cells, reducing MHC-II, TNFa and ROS production in bone marrow derived macrophages	[117]
mir-486	Adult female ICR mice	upregulated	0, 1, 2, 3 and 7 days after SCI	Transection	T11	miR-486 targets NeuroD6 and reflects apoptosis	[147]
mir-96 mir146a	Adult female SD rats	upregulated	4 h, 1 day, and 7 days after SCI	Contusive	T10	apoptosis through the concomitant increase in expression of the proapoptotic proteins caspase3	[105]
mir-107	Adult female SD rats	upregulated	4 h, 1 day, and 7 days after SCI	Contusive	T10	Apoptosis	[105]
mir-1	Adult female SD rats	upregulation	4 h, 1 day, and 7 days after SCI	Contusive	T10	Inflammation, oxidation and apoptosis	[105]
mir-129	Male Sprague Dawley rats	downregulated	4 and 14 d after SCI	Contusive	T12–T13	cell cycle, cell proliferation, cell differentiation	[124]

## Abbreviations

SCI	Spinal cord injury
CS	Cervical spine
TS	Thoracic spine
LS	Lumbar spine
BSB	Blood–Spinal–Barrier
ICU	Intensive care unit
